# Supplementary material for: Surface Microbiome Profiling of Dental Elevators Using Third‐Generation Sequencing: Implications for Infection Control in Dental Practice
Source: Microbiologyopen. 2025 Dec 8;14(6):e70178. doi: 10.1002/mbo3.70178 (PMC12685768; doi:10.1002/mbo3.70178)
Supplement: Supplementary file 1 — customer_backup. [file MBO3-14-e70178-s001.zip › customer_backup/customer_backup/function_analysis/KEGG/allsample/K2species/allsample.reabundance_Class2Order.txt.pdf]

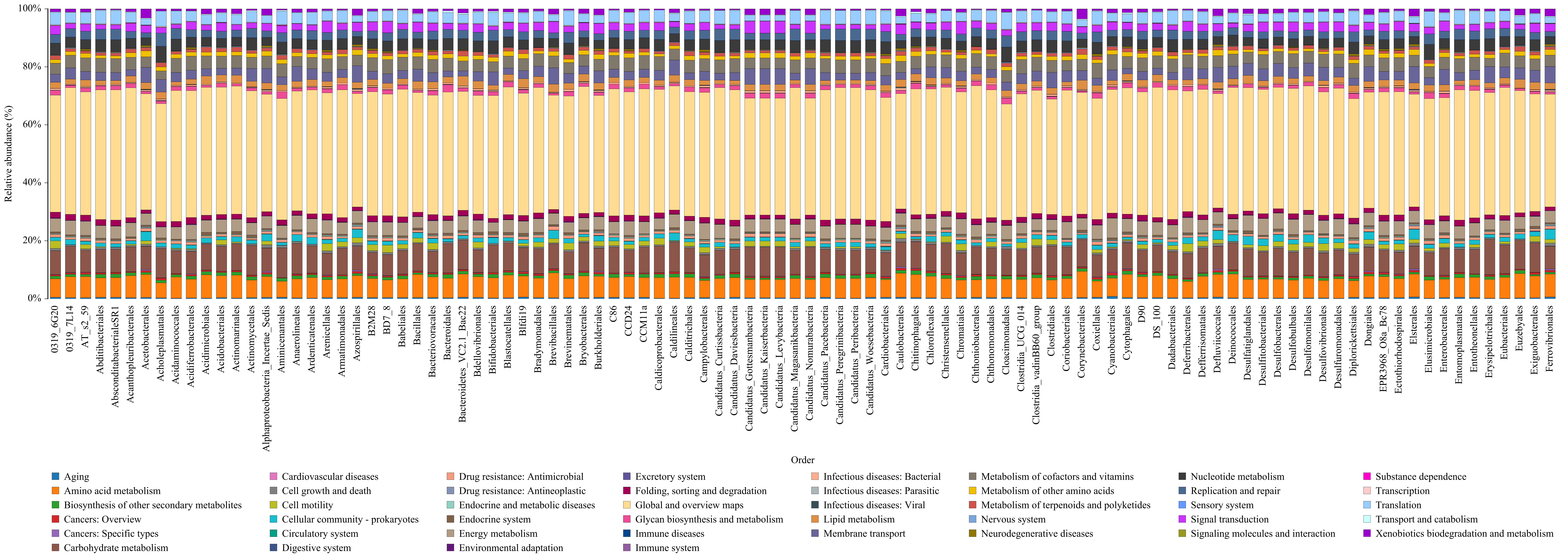

100%
80%
60%
40%
20%
0%
Relative abundance (%)
0319_6G20
0319_7L14
AT_s2_59
Abditibacteriales
AbsconditabacterialeSR1
Acanthopleuribacterales
Acetobacterales
Acholeplasmatales
Acidaminococcales
Acidiferrobacterales
Acidimicrobiales
Acidobacteriales
Actinomarinales
Actinomycetales
Alphaproteobacteria_Incertae_Sedis
Aminicenantales
Anaerolineales
Ardenticatenales
Arenicellales
Armatimonadales
Azospirillales
B2M28
BD7_8
Babeliales
Bacillales
Bacteriovoracales
Bacteroidales
Bacteroidetes_VC2.1_Bac22
Bdellovibrionales
Bifidobacteriales
Blastocatellales
Blfdi19
Bradymonadales
Brevibacillales
Brevinematales
Bryobacterales
Burkholderiales
C86
CCD24
CCM11a
Caldicoprobacterales
Caldilineales
Calditrichales
Campylobacterales
Candidatus_Curtissbacteria
Candidatus_Daviesbacteria
Candidatus_Gottesmanbacteria
Candidatus_Kaiserbacteria
Candidatus_Levybacteria
Candidatus_Magasanikbacteria
Candidatus_Nomurabacteria
Candidatus_Pacebacteria
Candidatus_Peregrinibacteria
Candidatus_Peribacteria
Candidatus_Woesebacteria
Cardiobacteriales
Caulobacterales
Chitinophagales
Chloroflexales
Christensenellales
Chromatiales
Chthoniobacterales
Chthonomonadales
Cloacimonadales
Clostridia_UCG_014
Clostridia_vadinBB60_group
Clostridiales
Coriobacteriales
Corynebacteriales
Coxiellales
Cyanobacteriales
Cytophagales
D90
DS_100
Dadabacteriales
Deferribacterales
Defferrisomatales
Defluviicoccales
Deinococcales
Desulfatiglandales
Desulfitobacteriales
Desulfobacterales
Desulfobulbales
Desulfomonilales
Desulfovibrionales
Desulfuromonadales
Diplorickettsiales
Dongiales
EPR3968_O8a_Bc78
Ectothiorhodospirales
Elsterales
Elusimicrobiales
Enterobacterales
Entomoplasmatales
Entotheonellales
Erysipelotrichales
Eubacteriales
Euzebyales
Exiguobacterales
Ferrovibrionales
Order
Aging
Amino acid metabolism
Biosynthesis of other secondary metabolites
Cancers: Overview
Cancers: Specific types
Carbohydrate metabolism
Cardiovascular diseases
Cell growth and death
Cell motility
Cellular community - prokaryotes
Circulatory system
Digestive system
Drug resistance: Antimicrobial
Drug resistance: Antineoplastic
Endocrine and metabolic diseases
Endocrine system
Energy metabolism
Environmental adaptation
Excretory system
Folding, sorting and degradation
Global and overview maps
Glycan biosynthesis and metabolism
Immune diseases
Immune system
Infectious diseases: Bacterial
Infectious diseases: Parasitic
Infectious diseases: Viral
Lipid metabolism
Membrane transport
Metabolism of cofactors and vitamins
Metabolism of other amino acids
Metabolism of terpenoids and polyketides
Nervous system
Neurodegenerative diseases
Nucleotide metabolism
Replication and repair
Sensory system
Signal transduction
Signaling molecules and interaction
Substance dependence
Transcription
Translation
Transport and catabolism
Xenobiotics biodegradation and metabolism
